# Dynamic responses of a damaged double Euler–Bernoulli beam traversed by a ‘phantom’ vehicle

**DOI:** 10.1002/stc.2933

**Published:** 2022-02-10

**Authors:** Rohit Chawla, Vikram Pakrashi

**Affiliations:** ^1^ UCD Centre for Mechanics, Dynamical Systems and Risk Laboratory, School of Mechanical and Materials Engineering University College Dublin Dublin Ireland; ^2^ Marine and Renewable Energy Ireland (MaREI) Centre University College Dublin Dublin Ireland

**Keywords:** damage detection, double beam, double‐sided open crack, moving load, tuned mass damper

## Abstract

In this paper, the dynamic response of a damaged double‐beam system traversed by a moving load is studied, including passive control using multiple tuned mass dampers. The double‐beam system is composed of two homogeneous isotropic Euler–Bernoulli beams connected by a viscoelastic layer. The damaged upper beam is simulated using a double‐sided open crack replaced by an equivalent rotational spring between two beam segments, and the lower primary beam is subjected to a moving load. The load is represented by a moving Dirac delta function and by a quarter car model, respectively. Road surface roughness (RSR) is classified as per ISO 8606:1995(E). The effect of vehicle speed of the moving oscillator and variable RSR profiles on the dynamics of this damaged double Euler–Bernoulli beam system for different crack‐depth ratios (CDRs) at various crack locations is studied. It is observed that coupling of two beams leads to a vehicular effect on the damaged beam, even when no vehicle on it is present. The effects of single and multiple tuned mass dampers to control the vibrational responses of the primary beam due to damage on the secondary beam is studied next. The performance of tuned mass dampers to reduce the transverse vibrations of the damaged double‐beam system and of the quarter car is investigated. The paper links the coupling between the two levels of double beam with the inertial coupling of the vehicle to the double‐beam system.

## 1 INTRODUCTION

The double‐beam system in general has been an interesting problem for dynamicists and engineers.^1^, ^2^, ^3^, ^4^ This interest lies in the dynamics and its applications present due to the coupling of the two beams, which often give rise to new behaviours of applications. The typical model for such a system is composed of two elastically connected beams, which are (usually Euler–Bernoulli). The two beams are connected by spring–dashpot elements, distributed continuously along the length of the two beams, thereby coupling them through stiffness. This canonical model has been applied to represent a wide range of systems, including double‐walled carbon nanotubes, oil conveying composite pipes, double‐sheathing cable system, double‐beam cranes, double‐beam spectrometers and interferometers, continuous dynamic vibration absorber systems and floating slab railway tracks. Amongst them, applications of the model in the transportation sector has been historically popular.

Oniszczuk^5^, ^6^ studied the free and forced transverse vibrations of a vibrating system comprising of two slender, prismatic and homogenous beams joined by a Winkler elastic layer. A similar approach was conducted by Oniszczuk^7^, ^8^, ^9^ to model a one‐dimensional continuous system resistant to tension using a double string system connected by a Winkler elastic layer to study the free and forced vibrations, with and without damping, by implementing Bernoulli–Fourier method. Similarly, homogenous Winkler‐type elastic continuous systems have been used to model double plates, double membrane and composite systems comprising of single beam and a string by Oniszczuk.^10^, ^11^, ^12^, ^13^ Abu‐Hilal^14^, ^15^ derived a closed analytical solution of a single‐ and a double‐beam system comprising two elastic isotropic Euler–Bernoulli beams traversed by a moving load and studied its dynamics for different speed parameter, damping ratio and stiffness parameter using Green's function. Fei et al^16^ used a double‐beam system with a viscoelastic layer to model a double‐layer sheathing cable system and studied its dynamics using extended dynamic stiffness method (EDSM). Han et al^17^ studied the dynamic stiffness matrix (DSM) and the frequency equation of a double‐beam system using an improved Wittrick Williams algorithm. Li et al^18^ studied the dynamics of a viscoelastic double‐beam system with the lower beam connected to a rigid base below via a Winkler layer using modal expansion iterative method. A similar double‐beam system stacked on an elastic foundation was studied by Han et al,^19^ and the global DSM and the frequency equation were obtained for various boundary conditions subjected to an axial load. Vu et al^4^ proposed an exact method for solving a double‐beam system connected by means of a distributed spring–dashpot and subjected to external harmonic excitation at the midspan of the system. Buckling due to compressive axial loading on a double‐beam system was studied by Zhang et al.^20^ A combination of compressive axial load and forced transverse vibrations subjected on a double‐beam system with identical beam properties having a Winkler elastic layer was investigated by Zhang et al.^21^ A closed‐form analytical solution of an elastically connected double‐beam system using distributed transfer function with arbitrary linear density and flexural rigidity was studied by Liu and Yang.^22^ Jiang et al^23^ made a comparative study of an analytical exact solution and finite element (FE) model of a double‐beam system subjected to successive moving loads (four loads with different velocities). Recently, vibrational analysis of Timoshenko double‐beam system under compressive axial loading using Green's function has been investigated by Zhao et al.^24^ Deng et al^25^ performed a vibration and buckling analysis of double‐funtionally graded Timoshenko beam system coupled by a Winkler–Pasternak layer under axial loading using dynamic stiffness method. Double‐beam systems incorporating Rayleigh and Timoshenko beams considering rotatory inertia and shear, under compressive axial loading, has been studied by Stojanović et al.^26^ Stabilities and instabilities of a double‐beam system with a Winkler elastic layer, subjected to random forces, have been investigated in great detail using direct Lyapunov method by Pavlović et al.^27^ Several articles involving the investigation of the dynamics of a double‐beam system can be found in literature by Rezaiee‐Pajand and Hozhabrossadati,^28^ Hamada et al.,^1^ Rahman and Lee^29^ and Stojanović et al.^30^ Lateral vibrating beams coupled by spring–mass systems has been studied by Gürgöze and Erol.^31^ Analysis of a double‐rod set‐up coupled by a spring–damper system was modelled by Gürgöze and Erol.^32^ Dan et al^33^ studied complex composite cable systems using EDSM. Similarly, double‐beam systems has been used to model floating slab and embedded railway tracks by Hussein and Hunt^34^ and Shamalta and Metrikine.^35^

Despite these analyses, there is a significant paucity in literature in investigating such models for two‐level or double‐decker bridges and structural health monitoring and control aspects that are connected to such dynamics. For these types of bridges, the connected beams can accommodate traversing moving loads and their bending stiffness are connected by the continuous spring–dashpot system, which has a vertical stiffness. In reality, the vertical stiffness comes with some horizontal stiffness, which resists the vertical–horizontal shear pair effects (typically truss members). Consequently, there is an interpretation of the canonical double‐beam model, whose interpretation goes beyond typical viscoelastic railway track–subgrade interaction. For example, Takemiya and Bian^36^ studied how a double‐beam system representing rails on track and viaduct on pillars, respectively, by Winkler‐spring elements can be employed.

To better understand how such dynamics can be relevant for structural health monitoring and control for double‐decker bridges, it is important to model damage in such double‐beam systems, investigate effects of moving load for the coupled model in the presence of damage and also assess the impact of control considering both moving load and damage. Despite this clear need, such an investigation has not been considered. This paper attempts to bridge this gap.

Existing literature indicates that there are works that have considered certain aspects of this problem, but they are in a disjointed manner and without the impact of moving loads. Takahashi^37^ and Takahashi and Yoshioka^38^ presented a double‐beam model with follower force and estimated its modal properties with cracks, as has been by Li and Sun^39^ for arbitrary boundary conditions, similar to others^18^ with various solution methods.^40^, ^41^ Stojanović et al^30^ have provided closed‐form solutions for Timoshenko and Rayleigh models (Stojanović and Kozić^26^ and Zhao et al.^24^), as has been by Škec et al^42^ for brittle and quasi‐brittle surfaces. For composites, similar work exist (Liu and Yang^22^ and Rezaiee‐Pajand and Hozhabrossadati^28^), including buckling (Bochicchio et al^43^ and Deng et al.^25^). Stability of such systems due to random forces has been investigated by Pavlović et al.^27^ For non‐linear multilevel beams, a harmonic balance method^29^ has been observed to be effective. Bhatra and Maheshwari^44^ has modelled a double‐beam system for track–subgrade interaction^45^, ^46^ with moving loads. While Rezaiee‐Pajand et al^47^ indicate vibration suppression, it only looks into influence of system parameters on the free vibration response. Railway–subgrade has also been used by Mohammadzadeh et al^48^ for stochastic foundation under a moving load, as has been by Wu and Thompson^49^ for Timoshenko beam model. Zakęś and Śniady^50^ present a model of double‐beam solution. Three‐beam systems^51^ and similar layered beams^52^ have also been looked into. The use of viscoelstic layer^16^ for damping has also been investigated. Despite these works, the investigation of moving load with structural damage for a double‐beam system has not been considered in the literature.

In terms of structural control, unsurprisingly, passive damping has been considered for double‐beam systems. Oniszczuk^6^ investigated a static moving load on a double‐beam system and presented one of the beams as a vibration absorber. However, this does not consider the double beam as the main structure, where the control of vibration is required in a combined manner and where the relative stiffness between the upper and lower layer to transfer energy will not work, since one of the layers will register significantly higher vibrations. Under such circumstances, an independent absorber or multiple absorbers will be required, and its performance has not been investigated to date. Recently, Sanchez Gómez and Metrikine^53^ look into high rises and related double‐beam model, which also indicates the wide range of applications it can have.

For bridge‐like systems, the work by Abu‐Hilal^15^ remains the most obvious one for moving loads but ignores the inertial coupling which is fundamental to bridge–vehicle interaction or the interaction between a vehicle with a degree of freedom and the variation of the track under it in the form of surface roughness. Ignoring inertial coupling tends to feature in recent works by Jiang et al^23^ as well. On the other hand, several researchers (Lu et al.,^54^ Metrikine et al^55^ and Vostroukhov and Metrikine^56^) present this coupling in a rail–vehicle interaction model and investigate stability aspects but do not look into the damage or the control aspects.

Efforts in modelling damage in double‐beam system has been observed typically through Nguyen,^57^ but this has never taken moving load into account. For single‐beam systems, however, the use of moving load for damage detection is well documented (Cahill et al.,^58^ Law and Zhu,^59^ Mahmoud and Abou Zaid^60^ and Pakrashi et al.^61^) and even a rail damage–subgrade interaction by Yan et al^62^ model exists.

It is observed that the literature has not considered the possibility that the moving load can have on a damaged structure for a double‐beam model as a health monitoring tool. Moving loads are typically modelled as Dirac delta functions, and the sampling property of a Dirac delta function ensures that spatial changes like damage will not only be sampled in time history from such moving loads but also be filtered to both beams due to coupling effect. Under such circumstances, the damage information from one beam to another will be transferred, through a bridge–vehicle interaction, even though no vehicle is physically present on it. On the other hand, the undamaged beam can be loaded with a vehicle to probe the damage in the other beam through coupling, without physically requiring a vehicle on it. This ‘shadow’ effect or ‘phantom’ effect of moving vehicles is particularly interesting and useful in the context of monitoring, and there exists no work investigating and modelling it. This paper, as a first, models such damaged coupled beam interacting with moving vehicles, including inertial coupling and surface roughness, and investigates damage effects and detection possibilities. An elastically connected two identical Euler–Bernoulli beams has been used to represent a bridge with two decks (a bilevel bridge). The viscoelastic layer coupling the two beams is replaced by a spring–damper system whose coupling parameters like stiffness, damping and material properties can be changed. The upper beam is a cracked structural member with a double‐sided open crack, and its damage is simulated by an equivalent rotational spring (Narkis^63^) whose stiffness depends on various beam parameters and crack geometry. The dependence of natural frequency on the extent of damage within the structure is respected in the dynamics throughout all simulations.

Existing work on passive control of such systems is then extended by considering single and multiple tuned mass dampers (TMDs) to assess their performance for varied vehicular speed and damages. A tuning criterion has been shown here to perform well for different damage locations and extent. This investigation not only considers the control of both beams but also provides comparative control performance of the displacement, velocity and acceleration responses as a function of the level of coupling between the beams via damage parameter, along with the vehicular coupling aspects. Such a set of result is not investigated to date, and this paper provides a first and practical set of estimates, including the impact of varying mass ratios of the dampers.

This systematic numerical study provides the first model combining damage, vehicular effects and control for a wide range of practical scenarios, including road surface roughness (RSR), damage location and extent, TMD mass ratio and the effect of multiple TMDs. It then provides ideas of how to use such a model for bridge–vehicle interaction‐based structural health monitoring and provides performance estimates of TMDs under a wide variation of parameters. The first section models a damaged double‐beam system where the primary beam is subjected to a moving load. The second section follows the exact procedure incorporating a quarter car toy model. Surface roughness of the primary beam has been incorporated in this case, and the extent of the damage is studied here for various crack locations and different crack‐depth ratios (CDRs). A range of velocity parameter up to the critical velocity of the vehicle model is simulated. In the last section, a numerical study has been performed where single versus multiple TMDs with different mass ratios has been implemented as a passive control and a tuning criterion has been shown to reduce the overall vibrational responses of the double‐beam system for a varied velocity range and damage extent.

## 2 THEORY

### 2.1 Theoretical model of the damaged double‐beam system traversed by a moving load

**FIGURE 1:**
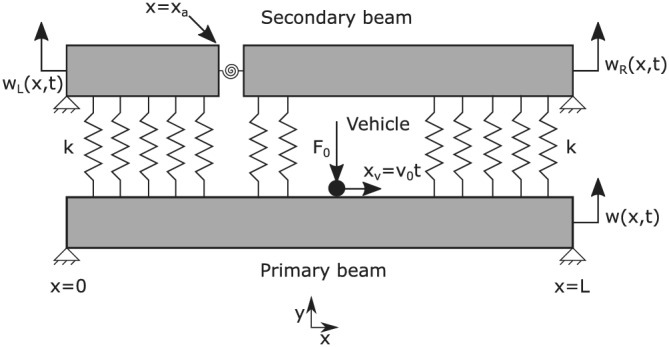
Double‐beam model with moving load

The double‐beam structure is modelled as two parallel Euler–Bernoulli beam placed on top of one another and coupled by a viscoelastic layer. Both the beams in this distributed spring–damper system has identical material properties (Appendix A in the Supporting Information): area of cross section *A*, geometric moment of inertia *I*, Young's modulus *E* and mass density *ρ*. The lower beam is designated as the primary beam and is subjected to a moving load represented by a moving Dirac delta function. The upper beam is considered to be a damaged structural member thereby weakening or ‘softening’ the entire double‐beam structure. It is assumed that the effect of the crack damage is local to its immediate neighbourhood and can be modelled as a rotational spring.^64^, ^65^ At the crack location, the damaged upper beam is divided into two separate subbeams connected by a rotational spring whose stiffness depends on the parameters of the beam and the crack geometry (Narkis^63^). Figure 1 shows the model considered in this paper.

### 2.2 Mathematical formulation for the double‐beam system

We consider two separate and identical Euler–Bernoulli beams^66^, ^67^, ^68^, ^69^ connected by a viscoelastic layer. The coupling between this double‐beam set‐up behaves as a spring damper system. The upper beam, described by *w*_*u*_(*x*, *t*), is divided into two subbeams and is separated at the crack location $x=x_{a}$. The transverse vibration of the subbeam to the left of the crack location is designated as *w*_*l*_(*x*, *t*), and the subbeam to the right is *w*_*r*_(*x*, *t*). The crack location is at a distance of *a* from the origin (left pinned end of a simply supported Euler–Bernoulli beam) as shown in Figure 1. The transverse vibration of the primary beam is described by *w*(*x*, *t*) given by Equation (1) with appropriate boundary conditions.

(1) $$\begin{array}{ll} w_{u} & =w_{l}(x,t)+w_{r}(x,t)\;0\leq x\leq L;\;w_{l}=w_{l}(x,t)\;0\leq x\leq a,\;\\ w_{r} & =w_{r}(x,t)\;\;\;\;a\leq x\leq L;\;w=w(x,t)\;0\leq x\leq L.\; \end{array}$$

The lower beam is subjected to a moving load *F*_0_, whose parameters (amplitude, velocity and inertial effects) can be controlled and changed accordingly. The double Euler–Bernoulli beam model are represented by Equation (2).

(2) $$\begin{array}{ll} EI\frac{\partial^{4}w_{u}}{\partial x^{4}} & +\mu \frac{\partial^{2}w_{u}}{\partial t^{2}}+k(w_{u}-w)+2\mu \omega_{b}\left(\frac{\partial w_{u}}{\partial t}-\frac{\partial w}{\partial t}\right)=0,\;\\ EI\frac{\partial^{4}w}{\partial x^{4}} & +\mu \frac{\partial^{2}w}{\partial t^{2}}+k(w-w_{u})+2\mu \omega_{b}\left(\frac{\partial w}{\partial t}-\frac{\partial w_{u}}{\partial t}\right)=F_{0}.\; \end{array}$$

The dynamics of the transverse vibrations of the beams are functions of space and time which need appropriate methods for solving. As per standard literature,^70^, ^71^ these vibrations are solved using the method of separation of variables or Bernoulli–Fourier method where the solutions are expanded as summation of modes shapes with a modal amplitude. One can interpret this summation to be an expansion of the solution in an orthogonal basis where the basis functions are the corresponding mode shapes. The coefficients in this expansion are the modal amplitudes that correspond to the temporal part of the solution. Therefore, the solution of Equation (2), using the method of separation of variables, is

(3) $$\begin{array}{ll} w_{u}(x,t) & =w_{l}(x,t)+w_{r}(x,t)=\sum_{n=1}^{\infty }\left(X_{ln}(x)+X_{rn}(x)\right)T_{un}(t)\;0\leq x\leq L,\;\\ w(x,t) & =\sum_{n=1}^{\infty }X_{n}(x)T_{ln}(t)\;0\leq x\leq L.\; \end{array}$$

The mode shapes *X*_*ln*_(*x*),  *X*_*rn*_(*x*) and *X*_*n*_(*x*) in Equation (3) is the homogeneous solution of the spatial part of an Euler–Bernoulli beam with no damping and stiffness (i.e., $\frac{\partial^{4}w_{n}}{\partial x^{4}}-\lambda_{n}^{4}\frac{\partial^{2}w_{n}}{\partial t^{2}}=0$, where $\lambda_{n}^{4}=\frac{\rho A}{EI}\omega_{n}^{2}$), that is,

(4) $$\begin{array}{ll} X_{ln}(x) & =A_{1}\sin(\lambda_{n}x)+A_{2}\cos(\lambda_{n}x)+A_{3}\sinh(\lambda_{n}x)+A_{4}\cosh(\lambda_{n}x)\;0\leq x\leq a,\;\\ X_{rn}(x) & =B_{1}\sin(\lambda_{n}x)+B_{2}\cos(\lambda_{n}x)+B_{3}\sinh(\lambda_{n}x)+B_{4}\cosh(\lambda_{n}x)\;a\leq x\leq L,\;\\ X_{n}(x) & =C_{1}\sin(\lambda_{n}x)+C_{2}\cos(\lambda_{n}x)+C_{3}\sinh(\lambda_{n}x)+C_{4}\cosh(\lambda_{n}x)\;0\leq x\leq L.\; \end{array}$$

The boundary conditions of a simply supported beam with no deflection or moment at x=0 and at x=L have been chosen. The damage in the structural member is due to the presence of a double‐sided open crack. It is assumed that the vertical deflection across the open crack is continuous. The influence of the structural damage in the secondary beam is local and affects only its immediate neighbourhood. Thus, at the crack location for the upper beam, the displacement, moment and shear forces at the crack junction are continuous. However, the slope of the displacement at the crack location is discontinuous. The crack is modelled by an equivalent rotational spring with stiffness being dependent on beam parameters, crack geometry and nondimensional crack flexibility, *θ* (Narkis^63^). The boundary conditions have been summed up as follows:

(5) $$\begin{array}{ll} w_{l}(0,t) & =0\;w_{r}(L,t)=0\;w(0,t)=0\;w(L,t)=0\;w_{l}(a,t)=w_{r}(a,t)\;w_{l}^{\prime \prime }(a,t)=w_{r}^{\prime \prime }(a,t),\;\\ w_{l}^{\prime \prime }(0,t) & =0\;w_{r}^{\prime \prime }(L,t)=0\;w^{\prime \prime }(0,t)=0\;w^{\prime \prime }(L,t)=0\;w_{l}^{\prime \prime \prime }(a,t)=w_{r}^{\prime \prime \prime }(a,t)\;w_{r}^{\prime }(a,t)-w_{l}^{\prime }(a,t)=L\theta w_{r}^{\prime \prime }(a,t).\; \end{array}$$

The nondimensional crack flexibility *θ* is a function of the CDR (a polynomial in *δ*) and is defined as^63^, ^72^, ^73^, ^74^

(6) $$\theta =6\pi \delta^{2}\frac{h}{L}(.5033-.9022\delta +3.412\delta^{2}-3.181\delta^{3}+5.793\delta^{4}),$$

where *h* is the height of the beam, *L* is the span of the beam and *δ* is the CDR.

Applying the boundary conditions Equation (5) on Equation (4) and solving for the coefficients of the modes shapes, that is, *A*_*i*_,  *B*_*i*_ and *C*_*i*_, we have

(7) $$\begin{array}{ll} X_{ln} & =A_{1}\sin\;(\lambda_{n}x)+\Delta A_{1}\sinh\;(\lambda_{n}x)\;0\leq x\leq a,\;\\ X_{rn} & =\delta A_{1}[\sin(\lambda_{n}x)+\alpha \;\cos(\lambda_{n}x)]+\gamma \Delta A_{1}[\sinh\;(\lambda_{n}x)+\beta \;\cosh\;(\lambda_{n}x)]\;a\leq x\leq L,\;\\ X_{n} & =C_{1}\sin\;(\lambda_{n}x)\;0\leq x\leq L,\; \end{array}$$

where Δ,  *δ* and *α* are functions of the beam length *L*, natural frequency *λ*_*n*_ and crack location *a* as

(8) $$\begin{array}{ll} \alpha & =-\frac{\sin\;(\lambda_{n}L)}{\cos\;(\lambda_{n}L)}\;\beta =-\frac{\sinh\;(\lambda_{n}L)}{\cosh\;(\lambda_{n}L)}\;\gamma =\frac{\sinh\;(\lambda_{n}a)}{\sinh\;(\lambda_{n}a)+\beta \;\cosh\;(\lambda_{n}a)}\;\delta =\frac{\sin\;(\lambda_{n}a)}{\sin\;(\lambda_{n}a)+\alpha \;\cos\;(\lambda_{n}a)}\;\\ \Delta & =\frac{\delta [\cos\;\lambda_{n}a-\alpha \;\sin\;\lambda_{n}a]-\cos\;\lambda_{n}a+L\theta \lambda_{n}\delta [\sin\;\lambda_{n}a-\alpha \;\cos\;\lambda_{n}a]}{\gamma [\cosh\;\lambda_{n}a+\beta \;\sinh\;\lambda_{n}a]-\cosh\;\lambda_{n}a-L\theta \lambda_{n}\gamma [\sinh\;\lambda_{n}a+\beta \;\cosh\;\lambda_{n}a]}.\; \end{array}$$

The natural frequencies for the mode shapes in Equation (7) are solved exactly by incorporating boundary conditions from Equation (5), in a matrix form (Appendix D in the Supporting Information) and solving for its determinant. For a non‐trivial set of solutions to exist, the determinant of the matrix at the natural frequency has to vanish. The natural frequencies have been numerically obtained and listed in Appendix B in the Supporting Information for various CDRs at different crack locations.

Once the mode shapes have been obtained from the boundary conditions, the dynamics of the modal amplitude (i.e., *T*_*un*_(*t*) and *T*_*ln*_(*t*)) are acquired, by substituting Equation (3) in Equation (2), multiplying the mode shape of the primary beam (i.e., *X*_*n*_(*x*)) using Equation (7), integrating from 0 to *L* and applying the orthogonality relationship $\int_{0}^{L}X_{(u,l)m}(x)X_{n}(x)dx=(L/2)\delta_{nm}$, as

(9) $$\begin{array}{ll} \mu C_{ul}\frac{d^{2}T_{un}}{dt^{2}} & +C_{ul}\left(EI\lambda_{n}^{4}+k\right)T_{un}-k\;C_{ll}T_{ln}+2\mu \omega_{b}\left(C_{ul}\frac{dT_{un}}{dt}-C_{ll}\frac{dT_{ln}}{dt}\right)=0,\;\\ \mu C_{ll}\frac{d^{2}T_{ln}}{dt^{2}} & +C_{ll}\left(EI\lambda_{n}^{4}+k\right)T_{ln}-k\;C_{ul}T_{un}+2\mu \omega_{b}\left(C_{ll}\frac{dT_{ln}}{dt}-C_{ul}\frac{dT_{un}}{dt}\right)=\int_{0}^{L}F_{0}X_{n}(x)dx,\; \end{array}$$

where the orthogonality integrals are defined as

(10) $$\begin{array}{ll} C_{ul} & =\int_{0}^{L}(X_{ln}(x)+X_{rn}(x))X_{n}(x)dx=\int_{0}^{a}X_{ln}(x)X_{n}(x)dx+\int_{a}^{L}X_{rn}(x)X_{n}(x)dx,\;\\ C_{ll} & =\int_{0}^{L}X_{n}^{2}(x)dx.\; \end{array}$$

The mode shapes in Equation (10) are composed of basis functions that are orthogonal and complete. However, the coefficients of mode shapes become functions of the CDR and the crack location due to incorporation of boundary conditions at the crack location. This results in an overshoot or undershoot of the basis functions and thus orthogonality is loosely satisfied. Thus, in order to preserve this orthogonality, the integrands in Equation (10) are determined numerically (Appendix B in the Supporting Information) using the boundary conditions given by Equation (5) and from Equation (8).

The forcing term representing the traversing load is defined by a moving Dirac delta function as

(11) $$F_{0}(x,t)=P_{0}\;\delta (x-x_{0}),$$

where $x_{0}=v_{0}t$ is the location of the load on the primary beam moving with a constant velocity *v*_0_.

## 3 NUMERICAL RESULTS

Numerical results of Equations (2) and (9) has been shown in this section. The mode shapes and the modal amplitude describing the spacial and temporal dependence of the transverse vibration for double‐beam model are discussed.

### 3.1 Mode shapes and natural frequency

Natural frequencies are numerically obtained by solving for the determinant of the coefficient matrix (Appendix D in the Supporting Information). The natural frequency of the double‐beam system depends on the crack location on the secondary beam and also on the extent of the damage at the crack location. The extent of the damage is defined by increasing the CDR. The natural frequencies of the system has been given in Appendix B in the Supporting Information for crack location a=0.05L,0.25L and 0.50*L* for varying CDR *δ* ranging from 0.05 to 0.35.

**FIGURE 2:**
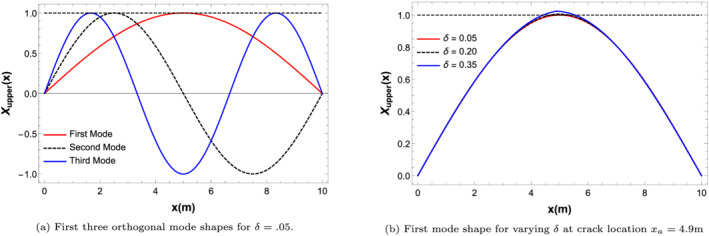
Mode shapes of the damaged secondary beam with a crack at $x_{a}=4.9$ m for varying CDRs (*δ*)

The mode shapes for the damaged secondary beam (Equation 7) has been shown in Figure 2 for various values of CDRs. The mode shapes for each natural frequency are seen to be orthogonal to one another. This property is frequently taken advantage of in most numerical analysis of beams and other structures.

**FIGURE 3:**
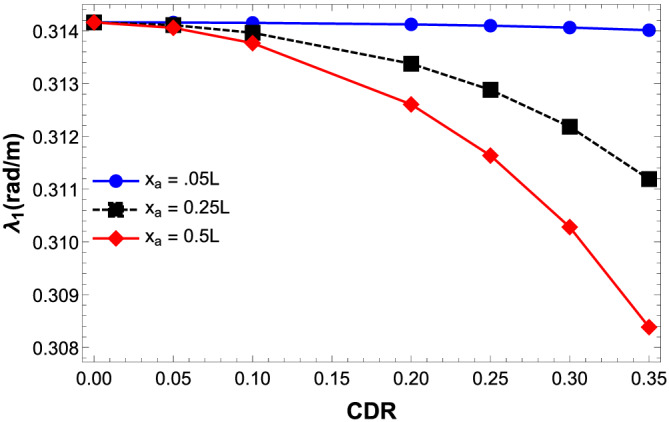
Natural frequency versus CDR at various crack locations

The dependence of damage on the natural frequency of the double‐beam model for various CDRs at different crack location is shown in Figure 3. It is observed that as the CDR increases, the system becomes more stiff and its natural frequency decreases. A similar effect is observed as the crack location comes near the midspan of the secondary damaged beam. Naturally, a central crack has more significant effect than an edge crack.

### 3.2 Importance of the first mode shape

**FIGURE 4:**
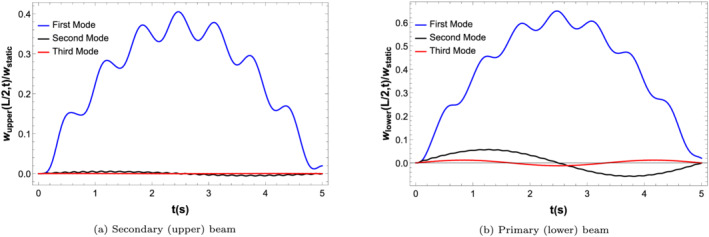
DAF for first three natural frequencies

Dynamic amplification factor (DAF) (Equation 12) for the first, second and third modes of the upper and lower beam coupled by a large stiffness value for an external load of 28*%* of the beam mass has been shown in Figure 4. Peak temporal response of the first modal amplitude has been compared to the second and third for both the beams. It is seen that for the upper beam, the second and third modal amplitude contributes to only 1.49*%* and 0.086*%* in comparison to the first modal amplitude while for the lower beam, the contributions are 8.8*%* and 1.86*%*, respectively. Thus, it is seen that only the first mode shape contributes to the transverse vibration of the beams. Therefore, vibrational responses of higher frequencies can be neglected.

### 3.3 Transverse vibration of primary and secondary beams

The transverse vibration of both the beams (i.e., Equation 3) is obtained numerically by multiplying the mode shape function from Equation (7) with the modal amplitude from Equation (9). The dynamical effect of moving load over the primary beam on both the beams is investigated. The concept of a ‘phantom’ vehicle is studied here. As the stiffness and damping of the viscoelastic layer connecting both the beams is increased, the transverse vibrations of the secondary beam increases due to the passage of a moving load on the primary beam. Although the secondary beam is not subjected to any forcing term, the coupling between the double‐beam system provided by the viscoelastic layer induces vibration due to vehicle movement on the lower beam. This is the concept of a ‘phantom’ vehicle.

**FIGURE 5:**
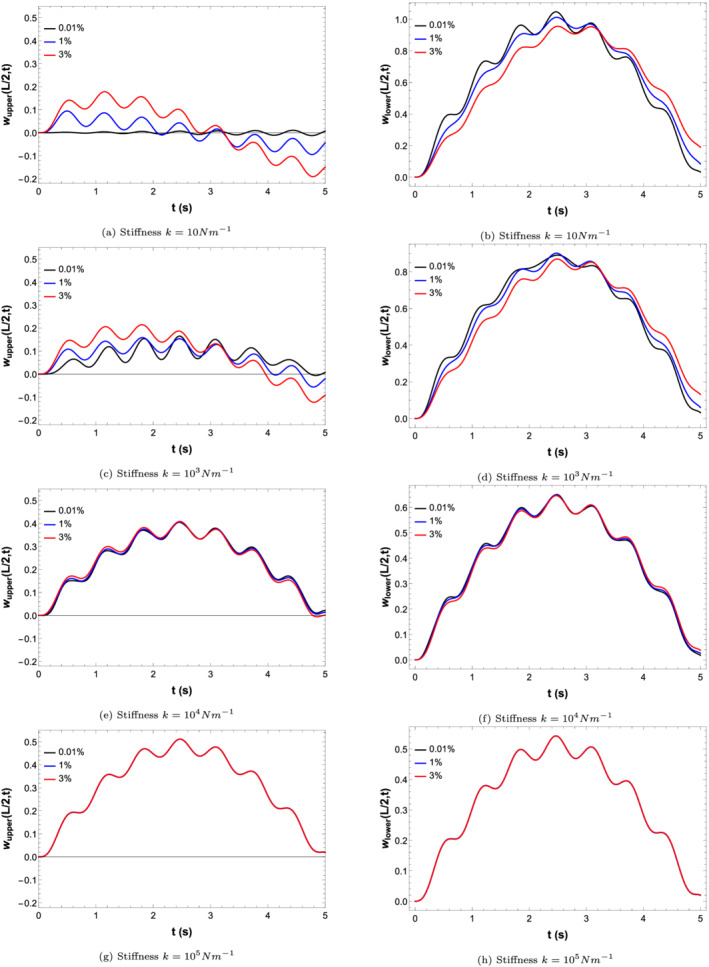
Normalised transverse vibrations of secondary beam (left) and primary beam (right) for varying stiffness (*k*) and damping coefficient of 0.01*%*,  1*%* and 3*%*

Figure 5 shows the normalised transverse vibrations of the double Euler–Bernoulli beam model for varying stiffness and damping factors. The extent of vibrations of the primary and secondary beams depends on the damping parameter and the stiffness of the viscoelastic layer between both the beams. Figure 5 shows that as stiffness increases, the transverse vibrations of the primary beam reduce as both the beams behave as a coupled system. When the beams are weakly coupled, the secondary beam vibrates 20*%* to that of the primary beam. Also the maximum displacement occurs when the load crosses the midspan of the primary beam. Increasing stiffness leads to more coupling between the double‐beam system, and for very high stiffness, the double‐beam model acts as a single rigid body.

For smaller values of stiffness *k* (i.e., when the double beam is weakly coupled), the absolute displacement for the upper and lower beam depend on the damping coefficient *c*. Smaller values of stiffness make the double‐beam system weakly coupled to each other. This leads to a weak elastic coupling between the primary and secondary beams. By increasing the damping coefficient, the secondary beam can be made to vibrate more. This is due to the fact that under weak elastic coupling, there is more transfer of mechanical energy from the primary beam to the secondary beam for higher values of damping coefficient. Thus, in case of weak elastic coupling, the damping coefficient provides coupling between the beams.

Figure 5 shows that for higher values of stiffness, the effect of damping is negligible. The coupling is provided directly by the stiffness of the viscoelastic layer, and the damping parameter does not contribute to the vibrational response. For a high value of stiffness, the double Euler–Bernoulli beam behaves as a single unit with maximum transfer of mechanical energy between the beams. The peak vibration of both the beams becomes roughly half of the static deflection (i.e., Equation 13).

**FIGURE 6:**
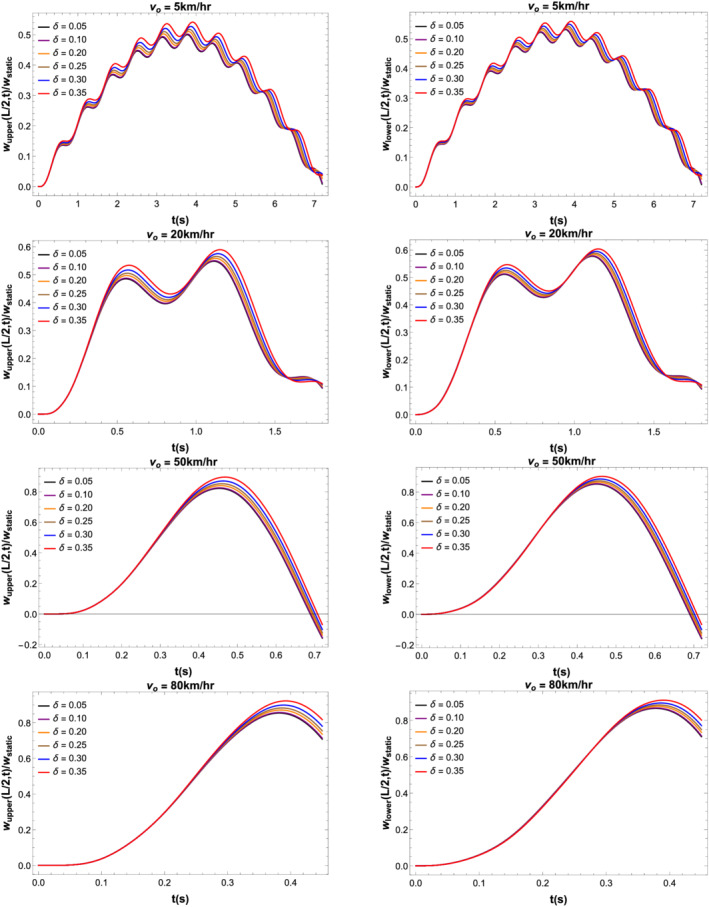
Transverse vibrational responses (normalised by static deflection at midspan) of the secondary beam (left) and primary beam (right) for varying CDRs *δ*

Figure 6 shows the vibrations of the strongly coupled primary and secondary beams with a large stiffness coefficient for various values of CDR when the crack location is at midspan of the beam. The system is excited by a load moving with velocities of 5, 20, 50 and 80 km/h. The effect of damage on the secondary beam increases vibrations of the entire beam structure. Although the primary beam is healthy, there is an increase in its vibrational response for increasing values of CDR. As the damage in the secondary beam increases, the beams vibrate more in comparison to the undamaged beam. Maximum vibration is observed when the moving load crosses midspan of the primary beam.

### 3.4 DAF and maximum displacement

DAF is a dimensionless quantity defined as the ratio of dynamic displacement of the beam to the static displacement of the beam when it is subjected to a static load at the midspan of the beam, that is,

(12) $$DAF=\frac{w_{dynamic}}{w_{static}}.$$

DAF describes how much factor should be multiplied to the transverse vibration caused by static load to obtain the transverse vibration due to dynamic forces in the system. Static deflection for an Euler–Bernoulli beam for a pinned–pinned support at both ends for a static load at midspan location is by

(13) $$F_{static}=\frac{F_{0}L^{3}}{48EI}.$$

**FIGURE 7:**
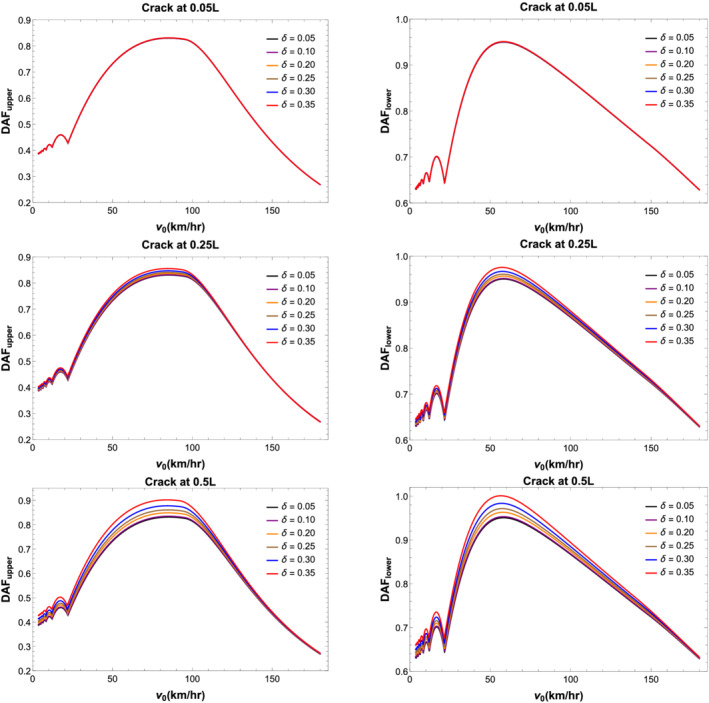
DAF versus velocity (km/h) of moving load for secondary (left) and primary (right) beams with different crack locations and increasing CDRs *δ*

DAF is computed by normalising the transverse vibrations of the beams (i.e., solving for Equation 2) with the static deflection for an Euler–Bernoulli beam (i.e., *F*_*static*_ Equation 13). The peak vibrational response of the primary and secondary beams at midspan is shown in Figure 7 for varying velocity, *v*_0_, of the moving load ranging from (0–180) km/h (up to critical velocity $\frac{\omega_{1}}{\lambda_{1}}∼175$ km/h). Figure 7 shows the DAF for different crack locations and with different values of CDR.

Maximum displacement, velocity and acceleration for the primary and secondary beams at the midspan are plotted for three different crack locations with increasing CDR against the velocity of the moving load, *v*_0_. The displacement is normalised against the maximum displacement for the primary beam with no damage when the vehicle crosses the midspan location with a velocity of 1 m/s. Velocity also has been normalised with maximum velocity for the undamaged primary beam while the acceleration of the beam has been normalised with g=9.8 m/s^2^.

**FIGURE 8:**
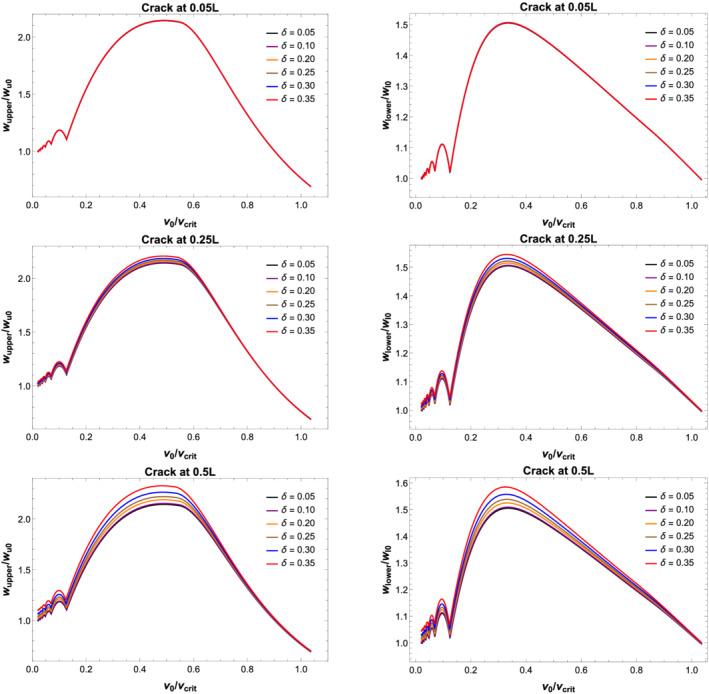
Normalised peak displacement versus velocity (km/h) of moving load for secondary (left) and primary (right) beams with different crack locations and increasing CDRs *δ*

Figures 7 and 8 show DAF and peak displacement of the beam element at the centre of the beams versus velocity of the moving load. For crack location near the edge of the secondary beam, the effect of the extent of damage (i.e., as the CDR increases) has minimum effect on the vibration of the beam element at the centre of the beam. As the location of the crack shifts to the centre of the beam, the maximum displacement of the beam element increases for larger damage. CDR, *δ*, of up to 0.35 has been plotted. Largest deflections occur in comparison to the undamaged primary beam when the crack location is at the midspan of the secondary beam. As the CDR increases from 0.05 to 0.35, the centre of both the primary beam and secondary beams vibrates more in comparison to the vibration of the same when the crack was located at the edge of the secondary beam. Evidently, the effect of damage on the beam element is most when the damage location is more towards the centre of the secondary beam. It is also seen that for very low speed of moving load, the DAF for both the beams is roughly half indicating the coupling nature of the system. As the velocity of the moving load increases, the DAF of both the beams is roughly unity indicating both the beams are vibrating up to the static deflection instead of half at lower velocities. Due to the viscoelastic coupling, the vibrations due to high velocities of the moving load are being distributed equally between both the beams.

**FIGURE 9:**
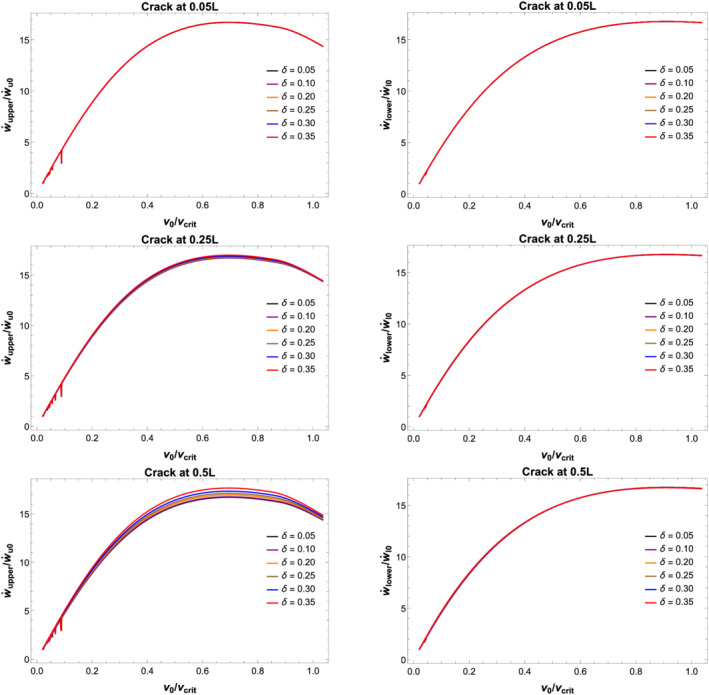
Normalised peak velocity versus velocity of moving load (km/h) for secondary (left) and primary (right) beams with different crack locations and increasing CDRs *δ*

**FIGURE 10:**
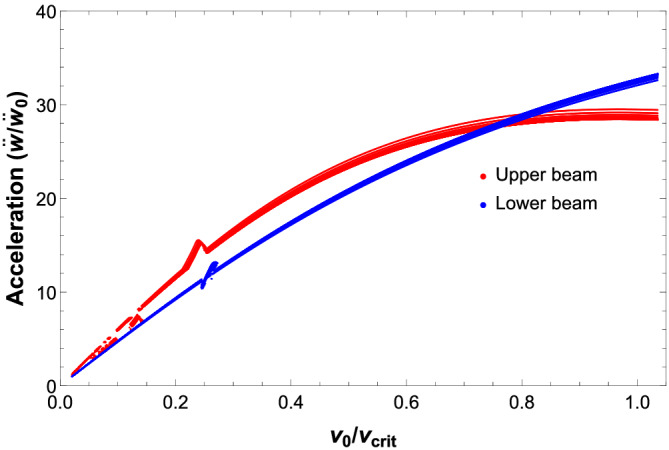
Normalised peak acceleration versus velocity (km/h) of moving load for all crack locations and increasing CDRs *δ*

Figures 9 and 10 plot normalised peak velocity and acceleration of the primary and secondary beam elements at the centre of the beam versus velocity of the moving load for various crack locations on the secondary beam with increasing CDRs. As previously discussed, the velocity of the beam element shows similar behaviour as the displacement for varying crack location and CDR. As the crack is located closer to the midspan of the secondary beam, the effect of increasing CDRs becomes apparent. For crack location a=0.5 m, increasing the CDR has minimum effect on the velocity of the beam element in comparison when the crack is located at the centre of the secondary beam, that is, a=4.9 m. However, there is minimum change in the acceleration of the beam element with varying crack location and CDR. Figure 10 plots the acceleration of the beam element of both the beams at the centre of the beam element for different crack location and CDR. It is apparent from Figure 10 that there is minimal change on the acceleration of the beam element due to the damage factors irrespective of its location.

## 4 DOUBLE‐BEAM SYSTEM WITH RSR PROFILES TRAVERSED BY A QUARTER CAR INCLUDING A TMD

### 4.1 Theoretical model

**FIGURE 11:**
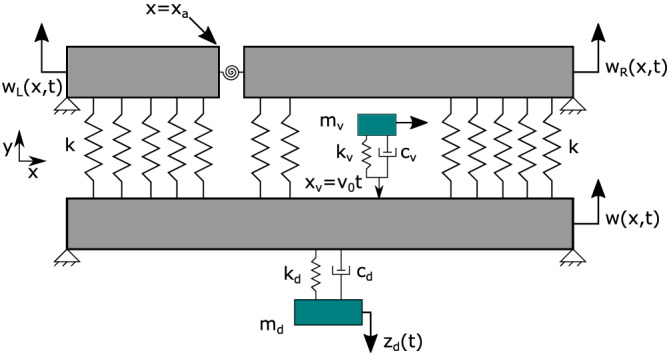
Double‐beam model with quarter car and tuned mass damper

The damaged double‐beam system considered in this section is identical to the model considered in previous section. The primary beam is subjected to a quarter car toy model with a single degree of freedom. It is assumed that the car is always in contact with the surface of the primary beam. The difference between the quarter car and a moving load is that it represents an oscillator that contributes small temporal fluctuations (inertial effect) about its own weight. The vehicle moves with a constant velocity with no acceleration and its location on the beam is described by the moving Dirac delta function. The upper beam designated as the secondary beam has an open crack which has been modelled similarly as a rotational spring. RSR is classified as per ISO 8606:1995(E).^75^ Different qualities of RSR profiles ranging from good to poor has been considered. Incorporation of multiple TMDs to reduce the effect of damage extent on the transverse vibrations of the system has been accounted for. The TMDs implemented in the model have been placed equidistant from each other below primary beam and a tuning criterion used by Pakrashi et al^76^ has been implemented to reduce the vibration response of the primary beam due to its coupling with the damaged secondary beam. The extent of damage has been represented by different CDRs. Figure 11 presents a model of this set‐up.

### 4.2 Mathematical formulation of the double‐beam system

The dynamical equations for the double‐beam system excited by a quarter car and controlled by a TMD or vibration absorber is given by Equations (14)–(16).

(14a) $$EI\frac{\partial^{4}w_{u}}{\partial x^{4}}+\mu \frac{\partial^{2}w_{u}}{\partial t^{2}}+k(w_{u}-w)+2\mu \omega_{b}\left(\frac{\partial w_{u}}{\partial t}-\frac{\partial w}{\partial t}\right)=0,$$

(14b) $$EI\frac{\partial^{4}w}{\partial x^{4}}+\mu \frac{\partial^{2}w}{\partial t^{2}}+k(w-w_{u})+2\mu \omega_{b}\left(\frac{\partial w}{\partial t}-\frac{\partial w_{u}}{\partial t}\right)=F_{v}\delta (x-x_{v})+\sum_{i=1}^{N}F_{di}\delta (x-x_{di}),$$

where *F*_*v*_ and *F*_*di*_ correspond to the external excitation force due to the vehicle and the *i*th TMD. The location of the vehicle on the primary beam and the TMDs are given by $x_{v}=v_{0}t$ and $x_{di}=x_{i}$ (placed equidistant below the primary beam). The dynamical equations of the vehicle and the *i*th TMD are

(15) $$m_{v}\frac{d^{2}z_{v}}{dt^{2}}+k_{v}{\left(z_{v}-w(x,t)-r(x)\right)}_{x=v_{0}t}+c_{v}{\left(\frac{dz_{v}}{dt}-\frac{\partial w(x,t)}{\partial t}-\frac{dr(x)}{dt}\right)}_{x=v_{0}t}=0,$$

(16) $$m_{d}\frac{d^{2}z_{di}}{dt^{2}}+k_{d}{\left(z_{di}-w(x,t)\right)}_{x=x_{i}}+c_{d}{\left(\frac{dz_{di}}{dt}-\frac{\partial w(x,t)}{\partial t}\right)}_{x=x_{i}}=0,$$

where *m*_*v*_,  *k*_*v*_,  *c*_*v*_ and *m*_*d*_,  *k*_*d*_,  *c*_*d*_ are the mass, stiffness and damping of the vehicle and the TMD. The RSR is given by $r(x_{v}=v_{0}t)$. The external excitation forces *F*_*v*_ and *F*_*di*_ are

(17) $$F_{v}=-m_{v}g+m_{v}\frac{d^{2}z_{v}(t)}{dt^{2}};\;F_{di}=-m_{d}\frac{d^{2}z_{di}}{dt^{2}}.$$

The solutions for the double‐beam system (Equation 14) is given by Equation (3). Applying the boundary conditions Equation (5) and the damage model Equation (6), we use the mode shapes Equation (7). The modal amplitude or time response of the beams are obtained by substituting Equation (3) in Equation (14), multiplying both sides with the mode shape of the primary beam (i.e., *X*_*n*_) and integrating from 0 to *L*. Using the orthogonality conditions (Equation 10) and the sifting property of the Dirac delta function, one obtains the temporal variations (*T*_*un*_(*t*) and *T*_*ln*_(*t*)) of the solution for the double‐beam system. Since only the first mode shape is significant in contributing to the transverse vibrations of the beam (Figure 4), dynamics of the modal amplitude for the first mode shape is given as follows:

(18a) $$\mu C_{ul}\frac{d^{2}T_{u}}{dt^{2}}+C_{ul}(EI\lambda_{1}^{4}+k)T_{u}-k\;C_{ll}T_{l}+2\mu \omega_{b}\left(C_{ul}\frac{dT_{u}}{dt}-C_{ll}\frac{dT_{l}}{dt}\right)=0,$$

(18b) $$\begin{array}{ll} \mu C_{ll}\frac{d^{2}T_{l}}{dt^{2}} & +C_{ll}(EI\lambda_{1}^{4}+k)T_{l}-k\;C_{ul}T_{u}+2\mu \omega_{b}\left(C_{ll}\frac{dT_{l}}{dt}-C_{ul}\frac{dT_{u}}{dt}\right)\;\\ & =\left(-m_{v}g+m_{v}\frac{d^{2}z_{v}}{dt^{2}}\right)\sin(\lambda_{1}v_{0}t)-\sum_{i=1}^{N}m_{d}\frac{d^{2}z_{di}}{dt^{2}}\sin(\lambda_{1}x_{i}),\; \end{array}$$

where *λ*_1_ is the natural frequency of the first mode shape and *C*_*ul*_ and *C*_*ll*_ are given by Equation (10). Expanding *w*(*x*, *t*) in Equations (15) and (16) and keeping the first mode shape, the dynamical equations for the vehicle and *i*th TMD are

(19) $$m_{v}\frac{d^{2}z_{v}}{dt^{2}}+k_{v}\left(z_{v}-\sin(\lambda_{1}v_{0}t)T_{l}-r(v_{0}t)\right)+c_{v}\left(\frac{dz_{v}}{dt}-\sin(\lambda_{1}v_{0}t)\frac{dT_{l}}{dt}-\frac{dr(v_{0}t)}{dt}\right)=0,$$

(20) $$m_{d}\frac{d^{2}z_{di}}{dt^{2}}+k_{d}\left(z_{di}-\sin(\lambda_{1}x_{i})T_{l}\right)+c_{d}\left(\frac{dz_{di}}{dt}-\sin(\lambda_{1}x_{i})\frac{dT_{l}}{dt}\right)=0.$$

The above system (Equations 18–20) is expressed in a matrix form and solved using the state‐space model. The matrices are given in Appendix C in the Supporting Information.

### 4.3 Road surface roughness

RSR used to generate a road surface profile for the primary beam has been classified as per ISO 8606:1995(E).^75^ The randomness of the profile is generated with a periodically modulated random process. In ISO 8606:1995 specifications, the surface roughness is a function of the vehicle velocity and is related by the velocity and displacement power spectral densities (PSD). The general form of the displacement PSD of RSR is given by

(21) $$S_{d}(f)=S_{d}(f_{0}){\left(f/f_{0}\right)}^{-\alpha },$$

where *f* is spatial frequency or discontinuity frequency in cycles/m and *f*_0_ is the reference spatial frequency and is equal to $\left(\frac{1}{2\pi }\right)$ cycles/m. *α* is an exponent for the PSD and for a vehicle with constant velocity, α=2. *S*_*d*_(*f*_0_) is the roughness coefficient in m^3^/cycle which describes the quality of the road. Five qualities of RSR profiles, Appendix A in the Supporting Information, have been studied here. From the PSD, a surface profile is generated by taking an inverse fast Fourier transform^77^, ^78^ and using Mathematica to generate random numbers, *ϕ*_*n*_ uniformly distributed between 0 and 2*π*. The RSR profile in its discrete form is given by^79^, ^80^, ^81^, ^82^, ^83^ and^84^

(22) $$r(\hat{x})=\sum_{n=1}^{N}\sqrt{4S_{d}(f_{0}){\left(\frac{2\pi n}{L_{c}f_{0}}\right)}^{-\alpha }\frac{2\pi }{L_{c}}}\cos\left(\frac{2\pi n}{L_{c}}\hat{x}+\phi_{n}\right),$$

where *N* is the number of discrete points on the surface profile, *L*_*c*_ is twice the length of the bridge and *ϕ*_*n*_ are random phase angles uniformly distributed between 0 and 2*π*.

### 4.4 Tuning parameters of the TMD

TMD or vibration dampers are used to reduce the vibrational responses of a system comprising a vibrating oscillator driven by an external force. For effective control of the vibrations of the primary system, due to excitations by the force, the parameters of the TMD have to be cleverly chosen or tuned. Parameters like natural frequency of both the primary system and the vibration absorber play an important role. Incorporation of one or many TMDs to control vibrational responses of systems exist in literature (Dinh and Basu^85^ and Wang et al.^86^). However, TMDs in bridges perform satisfactorily within a narrow region of allowed external frequency (Inman^87^) due to a wide bandwidth of moving loads. Implementation of single and multiple TMDs to improve the displacement responses of bridges due to single or many moving loads has been studied by several authors (Lin et al.,^88^ Wang et al.,^89^ Chen and Chen^90^ and Adam et al.^91^). Inclusion of damping in the system makes the analysis more complicated as higher value of damping may adversely effect the primary system resulting in detuning of the TMD. This is essentially an optimisation problem and needs to be numerically verified in multiple parameter space. In bridge engineering, there are limitations to the mass of the TMD which is attached to the primary vibrating structures. Typically, the mass ratios to control bridge vibrations range within (0.5–4)%. In systems with smaller mass ratios, the implementation of a single TMD with exact tuning is more rewarding. Since the first vibration mode provides maximum contribution to the dynamics, implementation of a TMD with optimal tuning parameter at the midspan of the beam (where maximum static deflection is observed) is more desirable (Warburton and Ayorinde^92^ and Law and Zhu^59^). Thus, a comparison has been made here between a single and multiple TMDs to control the responses of the double‐beam system, and the parameters have been tuned (based on fixed‐point theory by Den Hartog^93^) by a similar criterion proposed by Ghosh and Basu^94^ and Pakrashi et al.^76^

Equations for the primary beam and a single TMD located at the midspan (i.e., Equations 18 and 20) for N=1 can be rearranged as

(23a) $$\frac{d^{2}x}{dt^{2}}+\omega_{1}^{2}x+2\xi_{1}\omega_{1}\frac{dx}{dt}=\frac{f(t)}{m_{1}}+\mu_{0}(\omega_{d}^{2}u+2\xi_{d}\omega_{d}\dot{u})\sin^{2}(\lambda_{1}\frac{L}{2}),$$

(23b) $$\frac{d^{2}u}{dt^{2}}+\omega_{d}^{2}u+2\xi_{d}\omega_{d}\frac{du}{dt}=-\frac{d^{2}x}{dt^{2}},$$

where

$$\begin{array}{ll} & m_{1}=\mu C_{ll};f(t)=\left(k\;C_{ul}T_{u}+c\;C_{ul}\frac{dT_{u}}{dt}+(-m_{v}g+m_{v}\ddot{z_{v}})\sin(\lambda_{1}vt)\right)\sin(\lambda_{1}\frac{L}{2}),\;\\ & x=\sin(\lambda_{1}\frac{L}{2})T_{l};\;u=z_{d}-x;\;\omega_{1}=\sqrt{\frac{\left(EI\lambda_{1}^{4}+k\right)}{\mu }};\;\xi_{1}=\frac{c}{2\mu \omega_{1}};\;\mu_{0}=\frac{m_{d}}{m_{1}};\;\omega_{d}=\sqrt{\frac{k_{d}}{m_{d}}};\;\xi_{d}=\frac{c_{d}}{2m_{d}\omega_{d}}.\; \end{array}$$

Since $\lambda_{1}\approx (\frac{\pi }{L}),\;\sin(\lambda_{1}\frac{L}{2})\approx 1$, Equation (23) takes the form of the single degree of freedom system considered by Ghosh and Basu^94^ where the tuning parameters of the TMD system has been analytically derived using fixed‐point theory. Taking a Fourier transform of Equation (23), the transfer functions relating the amplitude of *x*(*t*) to the forcing term *f*(*t*) in the frequency domain (i.e., *X*(*ω*) and *F*(*ω*)) are

(24) $$X(\omega )=H_{x}(\omega )F(\omega )H_{x}(\omega )=\frac{H_{1}(\omega )}{m_{1}(1-\mu_{0}\omega^{2}H_{1}(\omega )H_{2}(\omega )(2i\xi_{2}\omega_{2}\omega +\omega_{2}^{2}))},$$

where *H*_1_(*ω*),  *H*_2_(*ω*) are

(25) $$H_{1}=\frac{1}{\sqrt{\omega_{1}^{2}-\omega^{2}+2i\xi_{1}\omega_{1}}};\;H_{2}=\frac{1}{\sqrt{\omega_{d}^{2}-\omega^{2}+2i\xi_{2}\omega_{d}}}.$$

Ghosh and Basu^94^ proposed that at the fixed‐point frequencies (frequencies at which the amplitude of *H*_*x*_(*ω*) is not dependent on damping of TMD), the peaks of *H*_*x*_(*ω*) are equal, thus minimising the responses of the primary structure. The values of *ω*_1_ and *ω*_*d*_ that satisfy this condition were solved analytically by Ghosh, and the optimum tuning frequency $\nu_{opt}=\frac{\omega_{d}}{\omega_{1}}$ has been implemented here where

(26) $$\nu_{opt}=\sqrt{\frac{1-4\xi_{1}^{2}-\mu_{0}(2\xi_{1}^{2}-1)}{{\left(1+\mu_{0}\right)}^{3}}}.$$

Expressing *ν*_*opt*_ in terms of partial fractions, followed by a binomial expansion, Pakrashi et al^76^ expressed *ν*_*opt*_ as

(27) $$\nu_{opt}=\sqrt{1-2\mu_{0}-4\xi_{1}^{2}+10\mu_{0}\xi_{1}^{2}}.$$

Further performing a binomial expansion reveals that *ν*_*opt*_ is linear in *μ*_0_ and quadratic in *ξ*_1_ neglecting higher order terms $O(\mu_{0}\xi_{1}^{2})$.

While choosing the damping parameter of the TMD, several values of damping has been simulated here to minimise the peaks of *H*_*x*_(*ω*). The chosen value of damping (i.e., *ξ*_*d*_) agrees with expressions for tuning the optimal damping ratio (*ξ*_*d*_) of a TMD available in literature. A simple expression has been shown to work effectively by Luft^95^ and has been verified and implemented here to control the transverse vibrations for this double‐beam set‐up.

(28) $$\xi_{d}=\frac{\sqrt{\mu_{0}}}{2}.$$

## 5 NUMERICAL RESULTS

### 5.1 Vibration analysis of double beam with a quarter car

Simulations for the transverse vibrations of the beams and vehicle motion were performed using the beam and vehicle parameters mentioned in Appendix A in the Supporting Information. The results have been found by evaluating the mean and root mean square of vibration responses of the primary and secondary beams for a range of vehicle velocities for different qualities of road surface profiles. The effects of damage at the crack location of the secondary beam for different CDRs on the vibration response of the double‐beam system has been studied.

**FIGURE 12:**
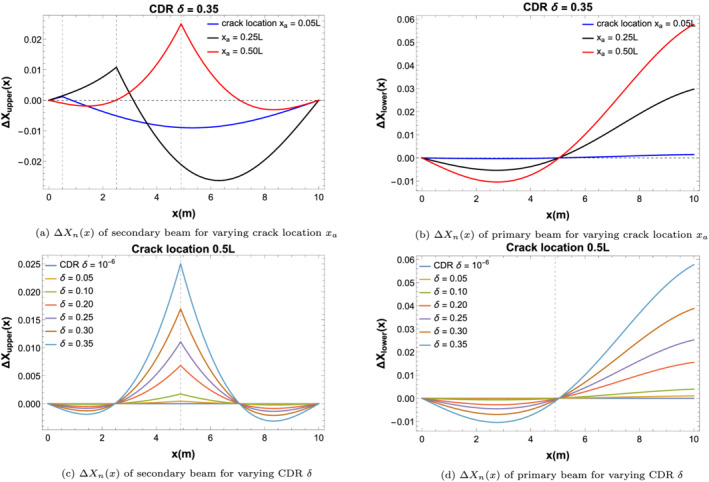
Mode shape difference function Δ*X*_*n*_(*x*) of primary and secondary beams for various crack locations *x*_*a*_ and varying CDRs *δ*

The mode shape difference function has been evaluated by calculating the difference between the mode shapes for the double‐beam system (i.e., Equation 7) and the mode shape for an undamaged beam (i.e., $\sin(\lambda_{n}x)$). Figure 12 shows the mode shape difference function for the primary and secondary beams at different crack locations with varying CDRs.

Figure 12 shows that there is a discontinuity in the slope of the mode shape difference function Δ*X*_*n*_(*x*) at the crack location in the secondary beam. The change in mode shape difference increases as the crack location moves towards the centre of the beam. For different CDRs, there is an increase in the mode shape difference function of the damaged secondary beam at the crack location. For the primary beam, there is an increase in the difference of the mode shapes as the crack location moves towards the centre of the secondary beam. The difference also increases as the CDR increases in the secondary beam. The damage is located in the secondary beam; however, due to both the beams being coupled to one another by means of the viscoelastic layer, the effects of the damage are reflected in the mode shape function of the primary undamaged beam.

**FIGURE 13:**
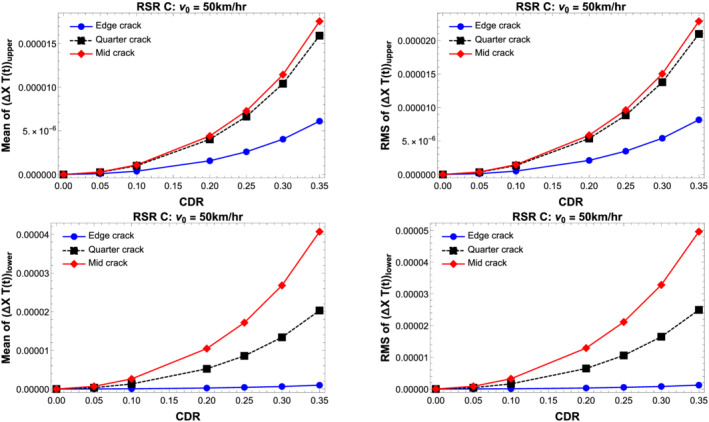
Mean and RMS of vibration response of Δ*X*_*n*_(*x*)*T*(*t*) for the double‐beam system for crack location $x_{a}=0.05L,\;0.25L$ and 0.5*L* and varying CDRs

Figure 13 shows the mean and root mean square of the vibration response of the mode shape difference function times the modal amplitude, that is, Δ*X*_*n*_(*x*)*T*_*n*_(*t*) for the primary beam and the secondary beam for varying CDRs and at different crack locations on the secondary beam. The velocity of the quarter car is chosen to be a moderate value of 50 km/h. The RSR profile used corresponds to an average quality of road surface. It is observed that for damage locations near the midspan of the secondary beam, the mean and RMS values increase along with increasing CDRs. The effect of the crack location on the vibration response is significant for the primary beam in comparison to the secondary beam although the damage is on the secondary beam. This is seen in the gradual increase on the mean and RMS values of both the beams as the crack location nears the centre of the beam. Hence, from now onwards, all the figures shown correspond to a damaged secondary beam with crack location at the midspan of the upper beam.

**FIGURE 14:**
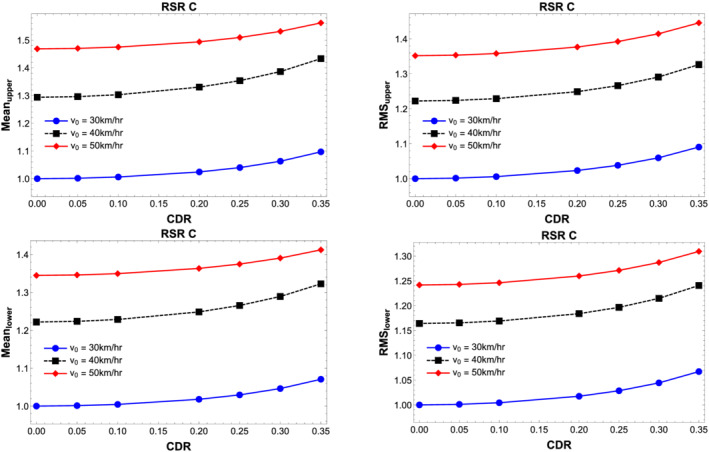
Normalised mean and RMS of vibration responses of the double‐beam system for quarter car velocity $v_{0}=30$, 40 and 50 km/h versus varying CDRs

Figure 14 shows the effect of vehicle velocity on mean and root mean square of the vibration responses of the beams. For lower velocities or very slow moving vehicles, the vibration response values are small in comparison to a medium vehicle velocity of around 50 km/h. This feature is also evident in Figure 8. A similar response is observed here. For higher velocities, the mean and RMS values decrease in comparison to the responses for medium range of velocities. This is because the vehicles do not spend much time in contact with the beam since the velocity is very high. In other words, there is not enough time for the vehicle oscillation to interact with the dynamics of the primary beam to perturb it. In Figure 14, the dynamics has been shown for three values of slow to medium vehicle velocity of 30, 40 and 50 km/h. It is clear that for increasing velocities, the mean and RMS values of the beam vibration increase with an increase in the CDR. Although the primary beam is not damaged and the secondary beam does not interact with the vehicle throughout the simulation, the vibrational responses of every beam element in the double‐beam set‐up increase with vehicle velocity and damage. If the difference of the transverse vibrations of the double‐beam model between damaged and undamaged secondary beams is known, then it is possible to calibrate the extent of damage from any given vibration sample and predict the location of damage with a qualitative idea as to the extent of the damage.

**FIGURE 15:**
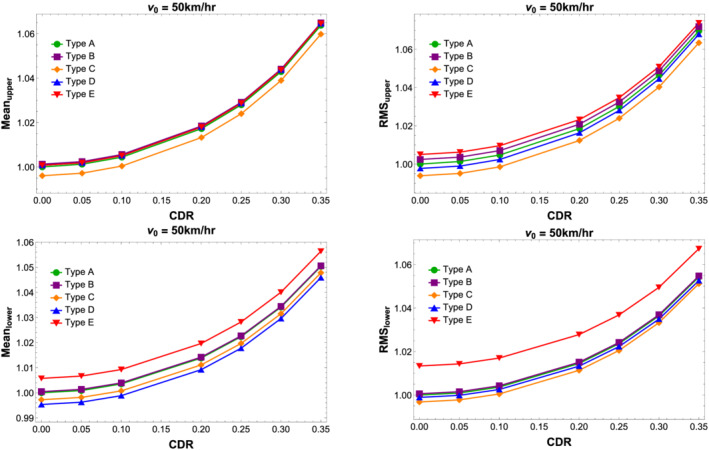
Normalised mean and RMS of temporal amplitude of the double‐beam system for varying quality of road surface profiles *S*_*d*_(*f*_0_) versus varying CDR

Figure 15 plots the mean and root mean square of the temporal amplitudes of the primary and secondary beams for different surface roughness profiles and varying CDRs. The vehicle velocity that provides the external excitation force considered for this case is $v_{0}=50$ km/h. The quality of the surface roughness is controlled by roughness coefficient *S*_*d*_(*f*_0_). Five different values of roughness coefficient has been chosen as mentioned in Appendix A in the Supporting Information. It is seen that for degrading quality of surface roughness, the vibrational response increases for larger CDRs.

**FIGURE 16:**
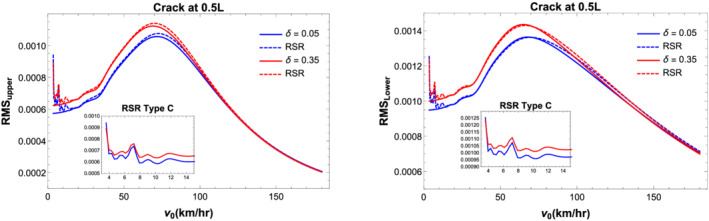
RMS of displacement response of secondary (left) and primary (right) beam with and without road surface profile for CDR, δ=0.05 and 0.35

Figure 16 plots the rms values of displacement at the midspan of primary and secondary beams for damaged and undamaged condition. Road surface profile of type C (Appendix A in the Supporting Information) has been incorporated. The figure shows fluctuations around the inertial coupling due to interaction of surface roughness for lower velocities of the quarter car. For higher velocities of the vehicle, these fluctuations are absent. However, the rms values increase with the inclusion of surface roughness for undamaged and damaged conditions of the secondary beam.

### 5.2 Vibration analysis of double beam with quarter car and TMD

Implementation of multiple TMDs is investigated in this section. The tuning parameters for the TMD is provided in Appendix A in the Supporting Information. Results are obtained by setting the mass ratio *μ* to 0.03 or 3*%* for every TMD while several values of the damping ratio of the TMD are investigated. For the optimal tuning frequency, Equation (24) has been used. The optimal tuning frequency is a function of mass ratio *μ* and the damping ratio of the primary beam, that is, *ξ*_1_. Since the damping ratio depends on the natural circular frequency of the double‐beam set‐up, when the CDR changes, the tuning frequency also needs to be changed for proper control of the vibration responses.

**FIGURE 17:**
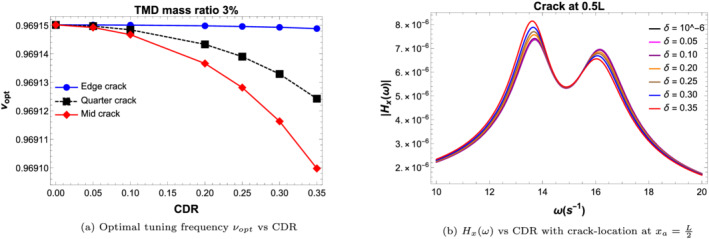
Effect of CDR on tuning parameters of TMD

Figure 17 shows the variation of the tuning frequency for varying CDRs at different crack locations. The variation of *ν*_*opt*_ is maximum for a crack location near the midspan of the secondary beam. This is because at that crack location, for the largest CDR considered here, the change in circular natural frequency is most pronounced. It is found that the change in the optimal tuning frequency *ν*_*opt*_ for increasing damage is not that significant. This is verified by studying peak values of the transfer function, Equation (24) with increasing damage at the midspan of the upper beam. For varying CDR, the amplitude of the transfer function at the fixed‐point frequencies is very similar and does not change significantly with changing natural frequency. This approves the result found previously by Pakrashi et al^76^ where for small values of *μ* and *ξ*_1_, their dependence on *ν*_*opt*_ is linear and quadratic. Since any change in *ξ*_1_ is negligible with change in CDR, the optimal tuning frequency does not change much. Therefore, we can use the same tuning parameters of an undamaged double beam to effectively control its vibrational response over a large span of time when damage is induced by external factors.

**FIGURE 18:**
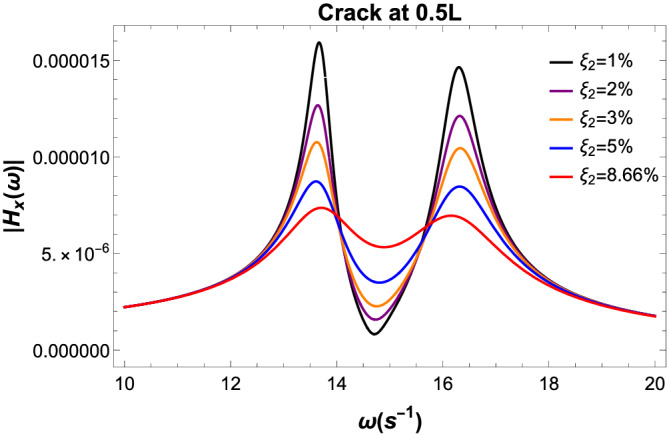
Transfer function for varying damping parameters of TMD

Figure 18 plots the variation of the transfer function for different damping ratios of the TMD used in the model. It is found that for increasing damping ratio for up to 8.66*%*, both the peaks of the transfer function at the fixed‐point frequency is similar and minimised. This value of the damping ratio *ξ*_*d*_ for a mass ratio of μ=3% effectively reduces the peak response of the transfer function as proposed by Luft.^95^ Hence, for all subsequent numerical analysis, the damping ratio of the TMD is taken to be $\xi_{d}=\frac{\sqrt{\mu }}{2}$.

**FIGURE 19:**
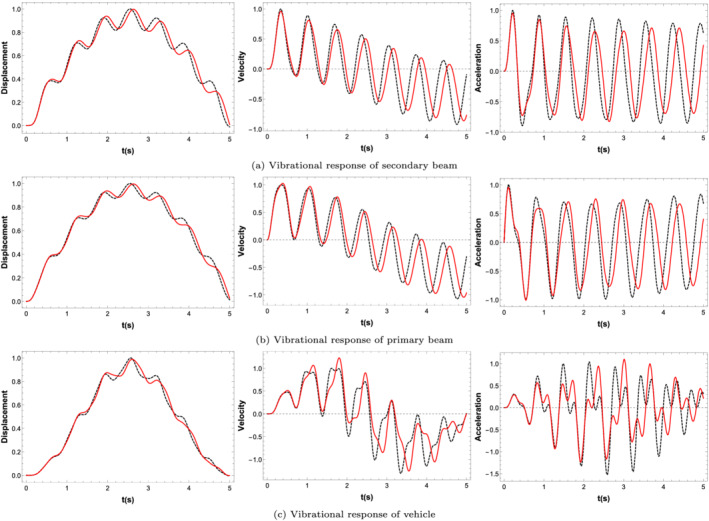
Normalised displacement, velocity, acceleration responses of primary beam, secondary beam and vehicle with and without TMD. Black dotted curves denote responses without TMD, and red curves denote control using a TMD

Figure 19 plots the normalised vibrational response of displacement, velocity and acceleration of the modal amplitude of the primary beam, secondary beam and the vehicle. A total of five TMDs located at regular intervals with one TMD always being at the midspan have been incorporated here, and all the TMDs have been tuned according to the parameters for an undamaged beam. For this figure, a vehicle is traversing the beam at a velocity of 10 km/h. The crack location is at the midspan of the secondary beam with a CDR of 0.35. The responses have been normalised with respect to the displacement, velocity and acceleration of the system without control using multiple TMDs. It is found that the tuned mass damper does not reduce the displacement of the primary and secondary beams for lower velocities of the vehicle. However, the TMD reduces the vibrational responses of the velocity and acceleration of the primary structure and vehicle. This feature is the importance of utilising TMDs to control the RMS responses of velocity and acceleration of the vehicle resulting in passenger comfort.

**FIGURE 20:**
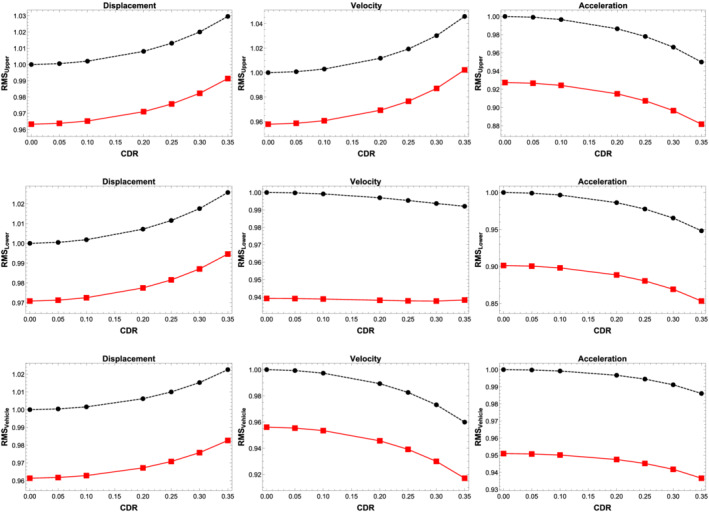
Normalised displacement, velocity, acceleration of primary beam, secondary beam and vehicle oscillation with and without TMDs for varying CDRs. Black dotted curve denotes responses without any TMD, and red curve denotes implementation of multiple TMDs

Figure 20 shows the normalised RMS values of the displacement, velocity and acceleration responses of the modal amplitude of primary beam, secondary beam and quarter car. The vehicle velocity is equal to 90 km/h. The figure shows a comparison of the RMS responses for varying CDRs with crack location at the midspan of the secondary beam with and without the usage of multiple TMDs. For this figure, five TMDs located at regular interval with a mass ratio of 3*%* have been used. The RMS values shown in the figure have been normalised against the RMS value corresponding to an undamaged beam. For varying CDRs, the TMDs effectively reduce the RMS responses. It is seen that the TMDs effectively controls the displacement, velocity and acceleration responses of the primary structure and the vehicle for every CDR considered. The TMDs has been tuned initially to the primary system with undamaged parameters. However, as the natural frequency of the primary structure changes with damage, the TMDs control the vibrations using the same optimal parameters of an undamaged beam. Thus, the optimal tuning frequency reduces the responses of the system with damage conditions as well.

**FIGURE 21:**
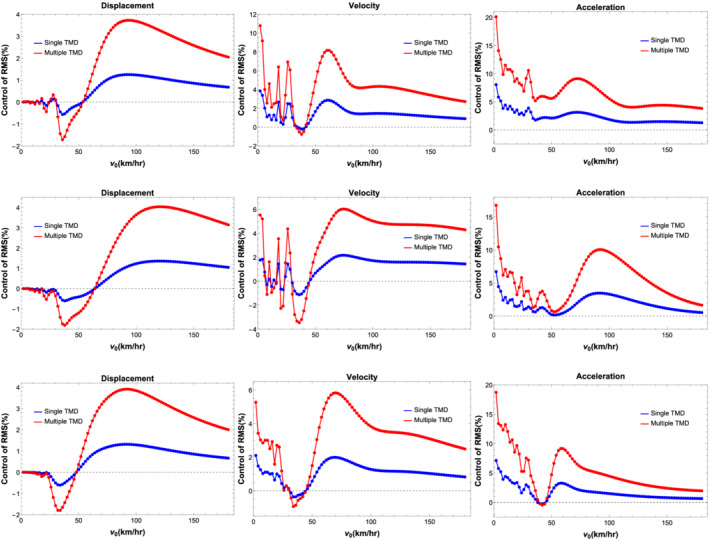
RMS control of displacement, velocity and acceleration for primary beam, secondary beam and quarter car

Figure 21 shows the percentage control of RMS values of displacement, velocity and acceleration of the double‐beam system and the quarter car for varied velocity range using a single and multiple TMDs with mass ratio μ=3%. Results for a single TMD located at the midspan of the primary beam versus multiple TMDs placed at regular intervals are shown. The plot refers to a crack location at the midspan with a CDR of 0.35. A wide range of velocities of the quarter car is studied. For different values of velocity, the TMDs tune and detune, highlighting the essential ranges for which the tuning criterion works. For lower range of velocities around (1–30) km/h, the control of RMS of displacement for the primary beam, secondary beam and the vehicle is not significant using either single or multiple TMDs. For velocity range of around (30–45) km/h, the TMDs detune in this region and adversely effect the displacement response by around 2*%*. The TMDs start performing better for higher range of velocity around (60–180) km/h where the displacement response control is around 4*%* using multiple TMDs and around 1*%* for a single TMD. The control of velocity responses of the system using TMDs is more desirable. For lower ranges of velocities around (1–30) km/h, there is better control of the velocity response in comparison to the displacement response using TMDs. Using multiple TMDs, the velocity response of the primary beam and the vehicle reduces by 6*%* while a single TMD located at the midspan reduces the response by 2*%* for higher velocity range. The performance of the TMDs is most effective in controlling the acceleration responses of the system and the vehicle. For lower velocities ranges around (1–50) km/h, the acceleration response reduces by 15*%* using multiple TMDs placed at regular intervals in comparison to around 10*%* when using a single TMD. For higher range of velocities (50–100) km/h, the control of acceleration response is around 10*%* using multiple TMDs. The figure also shows the performance of multiple TMDs versus a single TMD.

**FIGURE 22:**
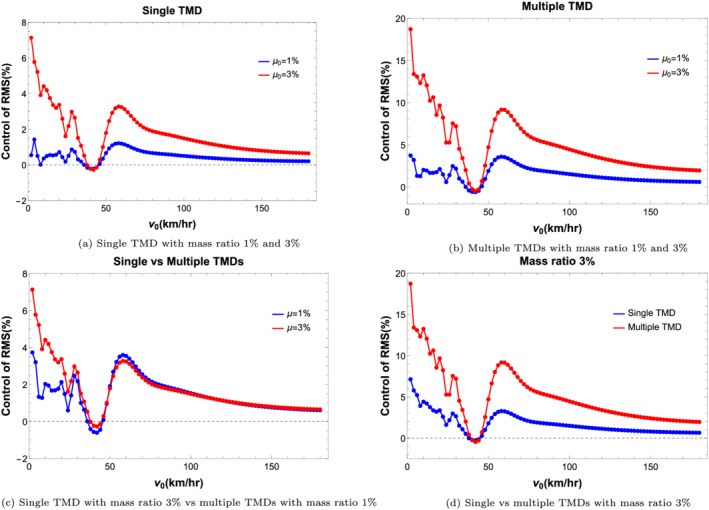
Control of RMS of acceleration response of quarter car with varying velocity for δ=0.35 at crack location $x_{a}=0.5L$

Figure 22 shows control of RMS response of quarter car acceleration using single and multiple TMDs having mass ratios of 1*%* and 3*%*. Figure 21a shows that a single TMD located at the midspan of the primary beam with higher mass ratio performs better. Figure 21b shows multiple TMDs with higher mass ratio performs better. Figure 21c shows that a single TMD with higher mass ratio of 3% performs better in comparison to multiple TMDs with lower mass ratio of 1*%*. Figure 21d shows that multiple TMDs perform better than a single TMD having the same mass ratio. However, in practical application, using multiple TMDs is not desirable due to physical constraints of the primary system. Thus, using a single TMD with a higher mass ratio with optimal tuning is more desirable.

**FIGURE 23:**
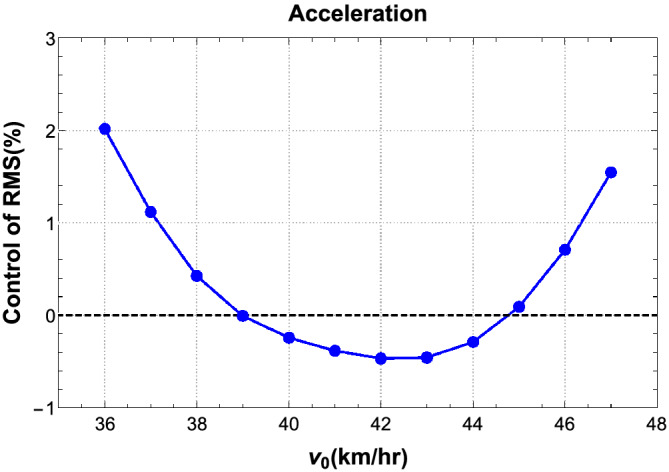
Detuning of TMDs in control of acceleration response of the vehicle

Figure 23 shows the velocity range where the TMDs are failing to control the acceleration response of the vehicle. In a narrow range of (38–45) km/h, the TMDs adversely effect the primary system and increase the responses by approximately 0.5*%*.

## 6 CONCLUSION

A systematic vibration analysis of a damaged double Euler–Bernoulli beam traversed by a moving quarter car is performed followed by a control analysis of the system using a TMD. The following conclusion are derived.

- The concept of a ‘phantom’ vehicle on damage detection and passive control is considered. The effect of damage at the crack location on the entire double beam indicates that as the extent of damage of the secondary beam increases, the stiffness of the entire system increases thereby resulting in the decrease in the natural circular frequency of the double‐beam set‐up. This results in an increase in the peak vibration responses of both the beams.
- The effect of coupling and stiffness reveals that for lower values of stiffness between the beams, that is, when the beams are under weak elastic coupling, there is lesser transfer of mechanical energy from the primary beam to the secondary beam. During such a set‐up, the damping coefficient of the viscoelastic layer between both the beams provides extra coupling between the double‐beam system. For higher values of stiffness, that is, when the primary beam is strongly coupled to the secondary damaged beam, there is a transfer of mechanical energy from the primary beam to the secondary; hence, as a result, the vibrations of both the beams increase. This leads to the idea of passage of a ghost or ‘phantom’ vehicle on the secondary beam even though no vehicle traverses it. The effect of damping coefficient on the dynamics of the double beam is negligible when the system is strongly coupled to one another. The effect of damage on the secondary beam (i.e., when the CDR is increased) leads to an increase in the vibration responses of the double‐beam system. Although the primary beam is healthy, the transverse vibration increases due to deterioration of health of the secondary beam.
- The dynamics of the system is studied for various velocity ranges of the vehicle. A surface roughness profile on the primary beam is incorporated. The extent of damage on the vibration responses for different damage conditions is studied. It is found that as the CDR in the secondary beam increases, the peak vibration of displacement response of both the beams increases. By choosing different surface profiles, the effect is studied in a similar fashion. Poor quality of surface roughness leads to an increase in the overall vibration response of the double‐beam set‐up.
- A comparative study between single and multiple TMDs has been carried out to control the vibrational responses of the damaged double‐beam set‐up. Control of RMS values of vibration responses of the system is studied for a wide range of vehicle velocity up to its critical speed. The tuning parameters for the TMDs to control the vibrational responses of the system have been chosen with respect to the parameters of the undamaged double‐beam set‐up. However, the same tuning criteria for single and multiple TMDs reduce the vibrational responses of the double‐beam set‐up for various damage conditions as well. For lower range of vehicle velocity, the TMDs control the velocity and acceleration of the vibrational responses of both the beams and the quarter car. For higher range of vehicle velocity, the TMDs attenuate the displacement responses of both the beams for every damage condition. It is found that multiple TMDs perform better to reduce vibrations in comparison to an implementation of a single TMD. However, due to physical restrictions of lower mass ratios of TMDs in most bridges, it is preferable to use a single TMD with a slightly higher mass ratio located at the midspan of the primary system rather than using a multiple TMD set‐up with lower mass ratio. Finally, it is found that the acceleration of the quarter car is also effectively attenuated after the implementation of single or multiple TMDs for a wide range of vehicle velocity. Thus, passenger comfort is improved using TMDs on the damaged double‐beam model.

## AUTHOR CONTRIBUTIONS

All authors contributed equally to the work.

## Supporting information



STC_2933‐sup‐0001‐Appendix.pdf

## Data Availability

All simulation data is available on request
