# Supplementary material for: Dynamic responses of a damaged double Euler–Bernoulli beam traversed by a ‘phantom’ vehicle
Source: Struct Control Health Monit. 2022 Feb 10;29(5):e2933. doi: 10.1002/stc.2933 (PMC9287083; doi:10.1002/stc.2933)
Supplement: Supplementary file 1 — STC_2933‐sup‐0001‐Appendix.pdf [file STC-29-0-s001.pdf]

Appendix A. Symbols and parameters

Table A.1: **Beam parameters**

Symbol	Parameter	Numerical Value	Units
E	Elasticity	10^{10}	Nm^{-2}
I	Area moment of inertia	6.75×10^{-4}	m^4
A	Cross-sectional area	9×10^{-2}	m^2
ρ	Density	8000	$kg\ m^{-3}$
L	Beam length	10	m
k	Stiffness of visco-elastic layer	10^5	Nm^{-1}
c	Damping coefficient	3%	$Nm^{-1}s$

Table A.2: **Vehicle parameters**

Symbol	Parameter	Numerical Value	Units
m_v	Body mass	1% of beam mass	kg
k_v	Stiffness	ω_v of $20s^{-1}$	Nm^{-1}
c_v	Damping coefficient	5%	$Nm^{-1}s$

Table A.3: **Road surface profiles**

Roughness Coefficient	A	B	C	D	E
$S_d(f_0)$	6×10^{-6}	16×10^{-6}	64×10^{-6}	216×10^{-6}	1024×10^{-6}

Table A.4: **Tuned damper parameters**

Symbol	Parameter	Numerical Value	Units
m_d	Body mass	$\mu = 3\%$	kg
k_d	Stiffness	ν_{opt}	Nm^{-1}
c_d	Damping coefficient	$\sqrt{\mu}/2$	$Nm^{-1}s$

Appendix B. Natural Frequency and Orthogonality

Damage within the structural model results in "softening" of the system. As a result the natural frequency of the doble-beam system decreases as the crack-location and crack-depth ratio is varied. Moreover orthogonality of the obtained mode shapes given by Eqs. (7) and Eqs. (8) in the manuscript and are numerically determined for different crack-locations and crack-depth ratios. Since the natural frequency is a function of damage parameters, the integrands in Eqs. (10) are numerically determined and given in the following tables.

Table B.5: **First mode shape frequency at different crack-locations for varying crack-depth ratios**

	CDR(δ)	10^{-6}	0.05	0.1	0.20	0.25	0.30	0.35
	Flexibility(θ)	10^{-13}	0.000659299	0.00251414	0.0100241	0.0163965	0.0254854	0.0385185
Crack location x_a (m)								
0.5	λ_1	.314159	.314157	.314150	.314121	.314096	.314061	.314011
2.5	λ_1	.314159	.314108	.313962	.313376	.312881	.312182	.311189
4.9	λ_1	.314159	.314056	.313766	.312605	.311636	.310280	.308384

Table B.6: **Orthogonal Coefficients**

Crack Location $x_a(m)$ $L = 10m$	CDR	C_{ul}	C_{ll}
$0.05L$	10^{-6}	4.9992	5.0
	0.05	4.99937	5.00004
	0.10	4.99738	5.00015
	0.20	4.98917	5.00061
	0.25	4.98209	5.00101
	0.30	4.97225	5.00156
	0.35	4.95826	5.00236
$0.25L$	10^{-6}	4.99999	5.0
	0.05	4.99891	5.00082
	0.10	4.99574	5.00314
	0.20	4.98314	5.01250
	0.25	4.97249	5.02043
	0.30	4.95757	5.03166
	0.35	4.93642	5.04770
$0.49L$	10^{-6}	5.0	5.0
	0.05	5.00156	5.00164
	0.10	5.00593	5.00627
	0.20	5.02322	5.02486
	0.25	5.03742	5.04047
	0.30	5.05697	5.06245
	0.35	5.08357	5.09343

Appendix C. State Space Model

The double-beam system along with the vehicle and tuned mass dampers are written in a matrix form and solved using the state space model. The numerics have been computed and verified in Matlab and Mathematica. The mass matrix, stiffness matrix and the state matrix are as follows,

$$\mathbf{M}\ddot{\mathbf{x}} + \mathbf{K}\mathbf{x} + \mathbf{C}\dot{\mathbf{x}} = \mathbf{F} \tag{C.1}$$

$$\dot{\mathbf{y}} = \mathbf{A}\mathbf{y} + \mathbf{B} \tag{C.2}$$

The vector $\mathbf{y}$ represents the system variables $(T_u, T_l, z_v, z_{d1}, z_{d2}, z_{d3}, ..., \dot{T_u}, \dot{T_l}, \dot{z_v}, \dot{z_{d1}}, \dot{z_{d2}}, \dot{z_{d3}}, ...)^{\mathbf{T}}$ along with,

$$\mathbf{M} = \begin{pmatrix} C_{ul}\rho A & 0 & 0 & 0 & 0 & 0 & 0 & 0 \\ 0 & C_{ll}\rho A & -m_v \sin(\lambda_1 vt) & m_d \sin(\lambda_1 x_1) & m_d \sin(\lambda_1 x_2) & . & . & . \\ 0 & 0 & m_v & 0 & 0 & 0 & 0 & 0 \\ 0 & 0 & 0 & m_d & 0 & 0 & 0 & 0 \\ 0 & 0 & 0 & 0 & m_d & 0 & 0 & 0 \\ 0 & 0 & 0 & 0 & 0 & . & 0 & 0 \\ 0 & 0 & 0 & 0 & 0 & 0 & . & 0 \\ 0 & 0 & 0 & 0 & 0 & 0 & 0 & . \end{pmatrix} \tag{C.3}$$

$$\mathbf{K} = \begin{pmatrix} C_{ul}(EI\lambda_1^4 + k) & -kC_{ll} & 0 & 0 & 0 & 0 & 0 & 0 \\ -kC_{ul} & C_{ll}(EI\lambda_1^4 + k) & 0 & 0 & 0 & 0 & 0 & 0 \\ 0 & -k_v \sin(\lambda_1 vt) & k_v & 0 & 0 & 0 & 0 & 0 \\ 0 & -k_d \sin(\lambda_1 x_1) & 0 & k_d & 0 & 0 & 0 & 0 \\ 0 & -k_d \sin(\lambda_1 x_2) & 0 & 0 & k_d & 0 & 0 & 0 \\ 0 & . & 0 & 0 & 0 & . & 0 & 0 \\ 0 & . & 0 & 0 & 0 & 0 & . & 0 \\ 0 & . & 0 & 0 & 0 & 0 & 0 & . \end{pmatrix} \tag{C.4}$$

$$\mathbf{C} = \begin{pmatrix} cC_{ul} & -cC_{ll} & 0 & 0 & 0 & 0 & 0 & 0 \\ -cC_{ul} & cC_{ll} & 0 & 0 & 0 & 0 & 0 & 0 \\ 0 & -c_v \sin(\lambda_1 vt) & c_v & 0 & 0 & 0 & 0 & 0 \\ 0 & -c_d \sin(\lambda_1 x_1) & 0 & c_d & 0 & 0 & 0 & 0 \\ 0 & -c_d \sin(\lambda_1 x_2) & 0 & 0 & c_d & 0 & 0 & 0 \\ 0 & . & 0 & 0 & 0 & . & 0 & 0 \\ 0 & . & 0 & 0 & 0 & 0 & . & 0 \\ 0 & . & 0 & 0 & 0 & 0 & 0 & . \end{pmatrix} \qquad \mathbf{F} = \begin{pmatrix} 0 \\ -m_v g \sin(\lambda_1 vt) \\ 0 \\ 0 \\ 0 \\ 0 \\ . \\ . \end{pmatrix} \tag{C.5}$$

$$\mathbf{A} = \left(\begin{array}{c|c} \mathbf{0} & \mathbf{I} \\ \hline -\mathbf{M}^{-1}\mathbf{K} & -\mathbf{M}^{-1}\mathbf{C} \end{array}\right) \qquad \mathbf{B} = \left(\begin{array}{c} \mathbf{0} \\ \hline \mathbf{M}^{-1}\mathbf{F} \end{array}\right) \tag{C.6}$$

Appendix D. Coefficient Matrix

$$\begin{pmatrix}
0 & 1 & 0 & 1 & 0 & 0 & 0 & 0 & 0 & 0 & 0 & 0 \\
0 & -1 & 0 & 1 & 0 & 0 & 0 & 0 & 0 & 0 & 0 & 0 \\
0 & 0 & 0 & 0 & \sin\lambda L & \cos\lambda L & \sinh\lambda L & \cosh\lambda L & 0 & 0 & 0 & 0 \\
0 & 0 & 0 & 0 & -\sin\lambda L & -\cos\lambda L & \sinh\lambda L & \cosh\lambda L & 0 & 0 & 0 & 0 \\
\sin\lambda a & \cos\lambda a & \sinh\lambda a & \cosh\lambda a & -\sin\lambda a & -\cos\lambda a & -\sinh\lambda a & -\cosh\lambda a & 0 & 0 & 0 & 0 \\
-\sin\lambda a & -\cos\lambda a & \sinh\lambda a & \cosh\lambda a & \sin\lambda a & \cos\lambda a & -\sinh\lambda a & -\cosh\lambda a & 0 & 0 & 0 & 0 \\
-\cos\lambda a & \sin\lambda a & \cosh\lambda a & \sinh\lambda a & \cos\lambda a & -\sin\lambda a & -\cosh\lambda a & -\sinh\lambda a & 0 & 0 & 0 & 0 \\
-\cos\lambda a & \sin\lambda a & -\cosh\lambda a & -\sinh\lambda a & \cos\lambda a + L\theta\lambda\sin\lambda a & -\sin\lambda a + L\theta\lambda\cos\lambda a & \cosh\lambda a - L\theta\lambda\sinh\lambda a & \sinh\lambda a - L\theta\lambda\cosh\lambda a & 0 & 0 & 0 & 0 \\
0 & 0 & 0 & 0 & 0 & 0 & 0 & 0 & 0 & 1 & 0 & 1 \\
0 & 0 & 0 & 0 & 0 & 0 & 0 & 0 & 0 & -1 & 0 & 1 \\
0 & 0 & 0 & 0 & 0 & 0 & 0 & 0 & \sin\lambda L & \cos\lambda L & \sinh\lambda L & \cosh\lambda L \\
0 & 0 & 0 & 0 & 0 & 0 & 0 & 0 & -\sin\lambda L & -\cos\lambda L & \sinh\lambda L & \cosh\lambda L
\end{pmatrix} \tag{D.1}$$
